# Detection of distal ureteral stones in pregnancy using transvaginal ultrasound

**DOI:** 10.1007/s40477-020-00504-4

**Published:** 2020-07-14

**Authors:** Michael S. Bold, James H. Boyum, Aaron M. Potretzke, Carl H. Rose, Thomas D. Atwell, Erik B. Sviggum, Brian C. Goss, Douglas L. Brown

**Affiliations:** 1 Department of Radiology, Mayo Clinic, 200 1st St SW, Rochester, MN 55902 USA; 2 Department of Urology, Mayo Clinic, Rochester, MN USA; 3 Department of OB/GYN and Maternal Fetal Medicine, Mayo Clinic, Rochester, MN USA; 4 Present Address: High Desert Radiology, Kingman, AZ USA

**Keywords:** Pregnancy, Ureterolithiasis, Transvaginal ultrasound, Renal colic

## Abstract

**Aims:**

To determine the performance of transvaginal ultrasound for the visualization of distal ureteral stones in pregnant patients with renal colic and to evaluate the diagnostic value of secondary findings suggestive of obstructing ureteral stone disease.

**Methods:**

We retrospectively identified 129 pregnant patients with a total of 142 encounters with both abdominal and transvaginal ultrasound. Ultrasound images for each patient were reviewed recording the presence of stone with location, hydronephrosis, resistive indices (RI), and status of the ureteral jets. Patients were subcategorized into two groups based on the visualization of distal ureteral stone.

**Results:**

The transvaginal technique identified 94% (*N* = 16/17) of sonographically detected stones in the distal ureter/urethra, while the transabdominal technique identified 29% (*N* = 5/17). The combined imaging for initial assessment of renal colic in pregnancy demonstrated a sensitivity of 89%, specificity 100%, and negative predictive value (NPV) of 98%. The frequency of hydronephrosis was statistically greater in the visualized stone group (94% vs 51%). Mean RI was identical in both groups however the delta RI was significantly elevated in those patients with distal ureteral stones with a mean delta RI value of 0.05. The rate of absence of ureteral jets was not statistically significant.

**Conclusion:**

The present data would suggest a utility of transvaginal ultrasound for the evaluation of the pregnant patient with 94% of distal stones being detected transvaginal versus 29% transabdominally. Additionally, there was significantly increased hydronephrosis and elevated RIs in patients with distal ureteral stones.

## Introduction

Urolithiasis affects approximately 1 in 11 people in the United States and is diagnosed with high sensitivity and specificity using non-contrast computed tomography (NCCT) [1]. In pregnant patients, renal colic is the leading non-obstetric cause of hospital admission, but the definitive diagnosis of urolithiasis in pregnancy is more difficult, complicated by the desire to avoid exposing the fetus to radiation from computed tomography (CT) [2–4]. As a result, ultrasound (US) has emerged as the first-line imaging modality to evaluate for stone disease in pregnancy [5].

Unfortunately, the performance of ultrasound in terms of sensitivity for ureteral stone detection has been suboptimal however most previous studies were often performed exclusively with transabdominal techniques. Viprakasit et al. assessed the utility of transabdominal US relative to NCCT. The authors reported sensitivity, specificity, and accuracy to be 40%, 84%, and 53%, respectively [6]. Other publications have similarly shown relatively disappointing sensitivities, ranging from 29 to 69% [5–8]. Sensitivity is even lower for direct stone detection of ureteral calculi; reported rates are as low as 15% when compared to NCCT [6].

Secondary US signs to indicate the presence of an obstructed ureter have been proposed and include: hydronephrosis, absent ureteral jets, and altered resistive indices (RI). Unfortunately, these parameters likewise do not perform well relative to CT and cannot differentiate the underlying cause of obstruction [4, 7]. This is particularly problematic in differentiating pathologic hydronephrosis from physiologic hydronephrosis of pregnancy, which has been reported to occur in up to 80% of pregnant patients [8]. Cognizant that direct visualization of a ureteral calculus allows a definitive diagnosis to be made. A few investigators have evaluated the ability to visualize ureteral calculi using transvaginal ultrasound (TVUS) revealing improved stone identification compared to transabdominal ultrasound (TAUS) alone [9, 10]. However, the reliability of a negative TVUS of the distal ureters in this setting has remained unclear [11].

The purpose of this study was to determine the performance of TVUS for the visualization of distal ureteral stones in pregnant patients presenting with renal colic. As a secondary aim, we evaluated the diagnostic value of secondary findings suggestive of obstructing ureteral disease, including hydronephrosis, absent ureteral jets, and altered resistive indices.

## Methods

This study was approved by our institutional review board and was compliant with the Health Insurance Portability and Accountability Act. Informed consent was waived; however, patients who denied the use of their healthcare information for research purposes were excluded from the study.

### Patient selection

A retrospective search was performed to identify all pregnant patients at our institution that underwent US evaluation for renal colic from April 4th, 2007 to November 18th, 2017. Patients were identified using our institutional Advanced Cohort Explorer, a clinical data retrieval tool, to help ensure all pregnant patients with suspected ureteral stone had been identified. The search terms included: pregnancy, renal colic, hematuria, hydronephrosis, stone, urolithiasis, ureterolithiasis, renal ultrasound, abdominal ultrasound, retroperitoneal ultrasound, pelvic ultrasound, and transvaginal ultrasound. Once the patients had been identified, an electronic medical record review was performed to abstract demographic and clinic history for each patient including age, pregnancy status, estimated gestational age, symptoms and their onset, and clinical outcome including conservative management and interventions. Patients, less than 18 years of age, and those who had indeterminate pregnancy status at the time of initial imaging evaluation were excluded.

Ultrasound imaging was performed using one of two imaging platforms (Acuson Sequoia, Siemens Medical Solutions; Logiq E9, GE Health Care). A standardized ultrasound scanning protocol for pregnant patients with suspected ureteral stones was used throughout the time period of this study. This standard protocol included assessment of the kidneys with an abdominal transducer to include multiple transverse and longitudinal views of each kidney. The RI was measured in interlobar or arcuate arteries of both the upper pole and the lower pole of each kidney. An abdominal transducer was used to image the bladder to assess the presence of a ureteral jet on each side. If the ureteral jet was not seen on the symptomatic side, the patient was rolled into the opposite decubitus position. While imaging the bladder, the sonographer attempted to identify the distal ureter, though it was often obscured by the presenting fetal part. Assuming no contraindications transvaginal imaging was then performed in an attempt to identify the distal ureter on each side of the bladder. Transvaginal scanning included transverse and sagittal views in region of the distal ureter, with sagittal views key to confirming that the stone was within the straight or gently curving tubular structure of the distal ureter (as opposed to a phlebolith that would have no identifiable, or a tortuous, surrounding tubular structure). A ureteral stone was considered present when an echogenic structure, regardless of acoustic shadowing, was identified in the distal ureter on either side. Given the frequent acuity of clinical presentation, the degree of bladder distension was not standardized however recommendations for a partially distended bladder or repeat scanning after rehydration were present in the scanning protocol.

The electronically stored static/cine images and radiologist interpretation for each patient encounter were reviewed to verify and record findings of stones, hydronephrosis, ureteral jets, and RI corresponding to the side of symptoms. The original interpretation, all made by board-certified radiologists, was used for these findings, though images were reviewed to ensure RI values were recorded for each patient. Stone was considered present when a focal, brightly echogenic, the structure was identified discretely in the kidney or in the ureter. The degree of hydronephrosis was graded qualitatively and then assigned a severity score to approximately quantitate the degree of dilatation (mild = 1, moderate = 2, and severe = 3). The severity score was averaged for each group. A ureteral jet was considered present if there was grayscale or color Doppler evidence of directional fluid motion from the ureteral orifice on either the transabdominal or transvaginal exams. The minimum and maximum resistive indices were recorded for each kidney and these values were then used to derive the group mean values. The mean RI of each kidney was compared to the mean of the contralateral kidney. The difference was recorded as the delta RI. Each ultrasound exam was considered an encounter, as some patients had more than one ultrasound exam during their evaluation for colic.

Patient follow-up included a review of the electronic medical record for clinical documentation of stone passage, collection and chemical analysis of stones, abnormal crystals/debris on urinalysis, and surgical interventions. Outpatient obstetric/urology clinical notes were reviewed throughout the pregnancy and early postpartum period for clinical documentation of confirmed/suspected stone passage and abnormal crystals/debris on urinalysis. Lastly, the Picture Archiving and Communication system (PACS) was reviewed for each patient for relevant subsequent imaging (ultrasound, MR, or CT) in the remainder of the pregnancy or postpartum period.

### Statistical analysis

Patients were considered as two cohorts based on sonographic absence or presence of distal ureteral/urethral stones. A ureteral stone was considered distal if it was in the distal 1/3 of the ureter. Demographic and outcomes data were compared between these two groups. The Chi-square test was used for binary categorical variables and the t-test for continuous variables. All p-values were two-sided, and p-value ≤ 0.05 was considered significant. The statistical package used was Social Science22 for Macintosh (IBM SPSS Statistics, Armonk, NY).

## Results

During the study period, 129 patients with a total of 142 encounters met inclusion criteria. The first group included 115 patients with a total of 125 negative encounters which are defined as no identifiable distal ureteral stone but included stones in the kidney and ureteropelvic junction (UPJ) (110 patients with a single encounter, 3 patients with 2 encounters, 1 patient with 4 encounters and 1 patient with 5 encounters). Gestational age at the time of each encounter was mean of 23.5 weeks, median of 26 weeks, minimum of 8 weeks, and maximum of 37 weeks. Sixty percent of the encounters had right-sided symptoms and 40% left-sided symptoms. The frequency of hydronephrosis was 51% with a scaled hydronephrosis severity of 1.5. The mean RI of the symptomatic kidney was 0.63 and delta RI 0.035. Ureteral jets on the symptomatic side were present in 57%. Sonography detected 20 intrarenal stones and UPJ stones in 5 patients. Two patients had UPJ stones missed by ultrasound, one confirmed by MRI and another by ureteroscopy. Two other patients had clinically documented the passage of a stone during hospitalization with recent preceding ultrasound findings in each patient showing intrarenal stones, moderate hydronephrosis and delta RIs of 0.06 in one patient and 0.1 in the other patient. Another separate patient had a report of passed ‘sludge’ which is nonspecific and could be seen with many entities including blood clots. Another separate patient had calcium oxalate crystals on urine microanalysis which has not correlated perfectly correlate with clinical stone disease.

**Fig.1:**
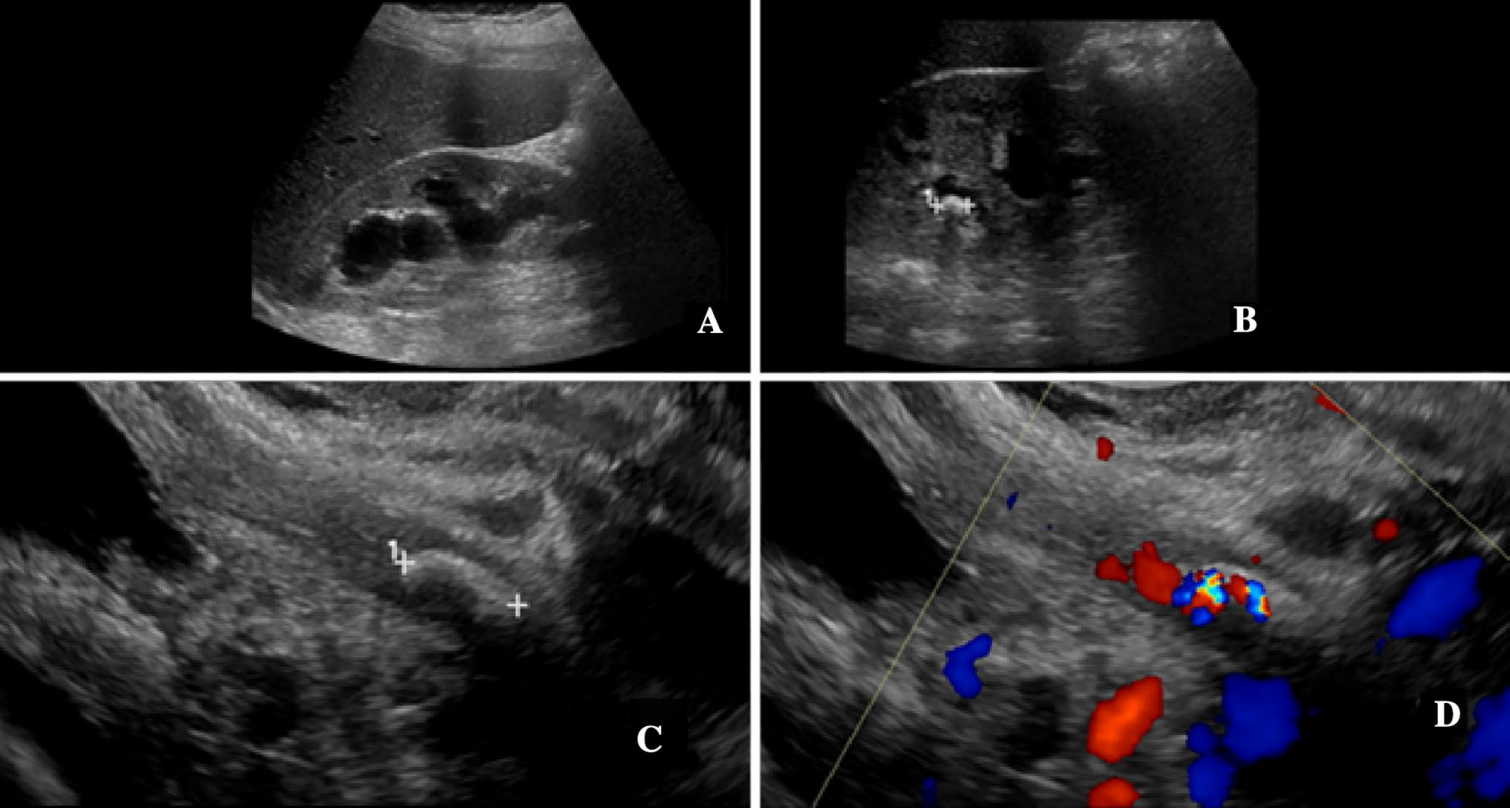
A 24-year-old G2P1001 presented at 30 weeks gestational age with right-sided renal colic. **a** Sagittal sonographic image of the right kidney demonstrated moderate hydronephrosis. **b** Additional sagittal image of right kidney shows an intrarenal stone, indicated by calipers. The transabdominal view of the bladder and ureterovesicular junction regions were reported negative. Subsequent sagittal transvaginal images in grayscale (**c**) and with color Doppler (**d**) demonstrate a stone (calipers) in the distal right ureter with twinkle artifact on color Doppler image. The patient was treated with cystoscopy, right ureteroscopy (which confirmed the stone), and stent placement. The patient carried the pregnancy to full term without complication

The second group of 16 patients with a total of 17 encounters had sonographically identified stones in the distal ureter (Fig. 1) or in the proximal urethra. As no ureteral stones superior to the distal ureter were identified in this study, other locations of ureteral stones were not evaluated. The group consisted of 14 unique patients with a single encounter, 1 patient with a separate negative encounter accounted for in the first group, and 1 patient with a distal ureter stone and a stone in the proximal urethra with subsequent negative encounter in pregnancies years apart. The stone identified in the proximal urethra by transvaginal ultrasound was included in the distal ureteral stone group for study purposes. Gestational age at the time of each encounter had a mean of 23.3 weeks, a median of 25 weeks, minimum of 4 weeks, and maximum of 38 weeks. 65% of the encounters had right-sided symptoms and 35% left-sided symptoms. The frequency of hydronephrosis was 94% with a scaled hydronephrosis severity of 1.5. The mean RI of the symptomatic kidney was 0.63 and delta RI 0.053. Ureteral jets on the symptomatic side were present in 31%. Comparison data between the two groups is presented in Table 1.

**Table 1:** Gestational age, hydronephrosis, ureteral jet, and RI comparison between groups

	Group 1 sonographically absent ureterolithiasis	Group 2 sonographically present ureterolithiasis	*p* value
Gestational age	23.5 weeks	24 weeks	
Hydronephrosis			
Frequency	51%	94%	*p* = 0.001
Average severity score	1.5	1.5	*p* ≥ 0.99
Present ureteral jets	57%	31%	*p* = 0.70
Resistive indices			
Mean	0.63	0.63	*p* ≥ 0.99
Delta	0.035	0.053	*p* = 0.03

TVUS identified 94% (*N* = 16/17) of sonographically identified stones in the distal ureter or urethra, while the TAUS identified 29% (*N* = 5/17) of distal ureteral stones. In only 1 case did the transabdominal technique identify a distal ureteral stone that the transvaginal technique did not. Of the patients with sonographically identified distal ureteral stones (*N* = 16) a ureteral stone was confirmed in ten patients. Five underwent procedural management with ureteroscopy (basket stone extraction, *N* = 3; and laser lithotripsy ± basket extraction, *N* = 2), five had clinically document spontaneous passage. The remaining six patients were lost to follow up.

In total, there were 142 combined transabdominal and transvaginal US encounters. 125 encounters had negative exams for direct distal stone visualization. In this cohort two encounters had missed urolithiasis confirmed by the passage; these will be considered false negatives though we do not know the location of the missed stone at the time of the ultrasound. Based on this retrospective data the combined exam had a sensitivity of 89.5%, specificity 100%, and negative predictive value of 98%. If there was no hydronephrosis, in addition to no ureteral stone being identified, no patients were found to have a missed stone on follow-up evaluation resulting in an NPV of 100%.

## Discussion

Women presenting with signs and symptoms of urinary stone disease in pregnancy can present a diagnostic challenge. The vast majority of pregnant women with ureteral stones have flank pain and/or hematuria [12]. Pregnant women with ureteral stones more commonly present in the second and third trimesters compared to the first trimester and most do not have a past history of stones [13, 14]. While the presence of flank pain at presentation seems no different in women with confirmed versus presumed stones, stones are less likely when hematuria is absent. The rate of spontaneous stone passage is probably less than reported in earlier studies (64–81%) and maybe closer to 48% [13].

In general, about 85% of ureteral stones in adults are in the distal ureter [10]. As transvaginal ultrasound can image the distal ureter in most female patients, it seems that such an imaging approach would be well suited for imaging distal ureteral stone disease [15]. The percent of ureteral stones located in the distal ureter in pregnancy however is uncertain.

Currently, the American College of Radiology (ACR) appropriateness criteria for acute flank pain with a suspected stone disease in pregnancy recommends ultrasound of the kidneys and bladder, with Doppler imaging as the most appropriate imaging test. CT and MRI are considered secondary modalities [16]. Transvaginal ultrasound is currently not specifically addressed in the ACR appropriateness guidelines. As transvaginal ultrasound usage becomes more common for obstetric and gynecologic applications this may change [17].

The current retrospective review using combined TAUS and TVUS imaging for initial evaluation of renal colic in pregnancy showed direct visualization of UPJ, distal ureteral and urethral stones in 85% of patients (*N* = 22/26; specifically 16 distal ureter stones, 1 urethra stone, 5 UPJ stones with 4 confirmed missed stones delineated in the group 1 results section), which is similar compared with 74% from a recent study [13]. Importantly, in this retrospective review, transvaginal ultrasound identified 94% of ureteral/urethral stones while the transabdominal ultrasound only identified 29%.

In the absence of direct stone detection with ultrasound, secondary sonographic signs of ureteral obstruction due to a stone can be utilized. However, the current data demonstrate the low specificity of these secondary indicators. For example, while hydronephrosis was statistically more common in the distal ureteral stone group (*p* = 0.001) the severity of hydronephrosis between the groups was nearly identical. Ureteral jets were the most unreliable indirect variable and likely related to hydration status, sonographer technique (location, scanning time, and patient position) and the known intrinsic variability in the normal population. Additionally, ureteral jets were identified in about a third of patients with distal ureteral stones, stressing the point that the presence of jets should not be used to exclude ureteral stones.

The mean RI for both groups was identical and similar to previously published data regardless of hydronephrosis or presence of stone [18, 19]. In the current study, however, the delta RI was significantly elevated in those patients with distal ureteral stones with a mean delta RI value of 0.05, falling in the range of previous studies predominantly reporting values of 0.04–0.08 [7]. Based on our review, an elevated delta RI should raise suspicion for an obstructive process.

We acknowledge there are limitations in the study. Although a defined protocol was used throughout the study, some inherent technical and patient-specific variation is inevitable over the 10-year duration of the study period. For example, hydration status and decubitus position are likely important for assessing ureteral jets in pregnancy [20, 21] and we were not able to ensure consistent methods were followed for either in this retrospective study. Additionally, a retrospective study is inherently prone to possible omissions in the medical record, including ultimate confirmation regarding the true presence of urolithiasis. For stones that were not seen on the US but later diagnosed based solely on the stone passage, we are unable to know where the stone was located at the time of the US; we were not able to determine if the stone was in the distal ureter and missed on the US or if the stone was located more superiorly in the ureter where bowel gas may have impeded identification. Additionally, of the 16 patients with transvaginal ultrasound detection of a distal ureteral stone, 6 were lost to follow-up; we considered these true positives as we think it very unlikely that an echogenic structure in the distal ureter would be falsely positive for a stone, but admit there is no independent confirmation of the ureteral stone.

### Conclusion

Our findings suggest that TVUS is useful for the evaluation of the pregnant patient with renal colic for determining the presence of a distal ureteral stone. Our data shows that TVUS identified 94% of detected distal ureter/urethral stone while TAUS detected 29%. Additionally, the combined TAUS/TVUS exam in pregnant patients presenting with renal colic had a sensitivity of 85% for all ureteral stones and an NPV of 100% for distal stones when no distal stone was identified and hydronephrosis was absent. When the initial abdominal ultrasound of the kidney and ureter is negative or inconclusive we advocate the routine additional use of the transvaginal technique for evaluation of the distal ureter.
